# Objectification of the Functional Myodiagnosis Muscle Test

**DOI:** 10.3390/jcm14155555

**Published:** 2025-08-06

**Authors:** Josef Franz Mahlknecht, Eugen Burtscher, Ivan Ramšak, Christine Zürcher, Johannes Bernard

**Affiliations:** 1 FMD Research Centre, 39030 Kiens, Italy; praxis@dr-mahlknecht.it; 2 IMAK/FMD, 6850 Dornbirn, Austria; eugen.burtscher@vol.at; 3 ICAK/International Diplomate Exam 2000/Goodheart, 9020 Klagenfurt, Austria; ramiv@gmx.at; 4 University Hospital for Conservative Dentistry and Periodontology, Medical University of Innsbruck, 6020 Innsbruck, Austria; christine.zuercher@i-med.ac.at; 5 IMAK/FMD, 6020 Innsbruck, Austria

**Keywords:** functional myodiagnosis, muscle test objectification, muscle test reliability, applied kinesiology

## Abstract

**Objective:** This study aimed to investigate whether the subjective assessments of strong and weak muscles in the Functional Myodiagnosis muscle test (FMD-MT) can be objectively and reproducibly verified using physically measurable parameters. Additionally, we sought to evaluate the reliability of the manual muscle test in order to reinforce the scientific evidence supporting this accepted, yet not widely adopted, complementary medicine method. **Methods:** In a crossover observational study, three experienced medical practitioners conducted the FMD-MT of the rectus femoris muscle on 24 healthy participants using a specially designed therapy bench, with all measurements recorded via an oscillogram. The study investigated the force–time integral, joint angle change, additional force load, mean force turning point 1, as well as the interrater reliability and validity of both examiner assessments and instrumental analyses for the two muscle reaction variants: strong and weak. **Results:** A significant difference between the response pattern of strong and weak muscles was identified for the force–time integral (*p* = 0.005), the change in joint angle (*p* < 0.001), and the additional force load (*p* = 0.001). No difference between strong and weak muscles could be detected regarding the force turning point 1 (*p* = 0.972). The examiners demonstrated 100% accuracy in identifying weak muscle reactions as weak, and 99.2% accuracy in identifying strong muscle reactions as strong (*p* = 0.316). The overall intra-class correlation coefficient was 0.984. The oscillogram correctly visualized weak muscle reactions in weak muscles with an accuracy of 81.7%, and strong muscle reactions in strong muscles with an accuracy of 86.7% (*p* = 0.289). **Conclusions:** The Functional Myodiagnosis muscle test (FMD-MT) enables a clear and objective differentiation between strong and weak muscles, with statistically significant differences observed in the force–time integral, additional force load, and joint angle changes. Under rigorously standardized testing conditions, the FMD-MT of the rectus femoris muscle demonstrates a validity rate of 99.6% and an excellent reliability (ICC 0.984). Consequently, the FMD muscle test proves to be a reliable, reproducible, and objective diagnostic method. Trial registration: German Register of Clinical Studies U1111-1212-6622.

## 1. Introduction

Functional Myodiagnosis (FMD) is a diagnostic method derived from Applied Kinesiology (AK) [1,2,3] and has been recognized by the Austrian Medical Association as a complementary medical procedure [4]. The Society for Functional Myodiagnosis (IMAK) [5] coined the term “Functional Myodiagnosis” and secured its legal protection. This term accurately encapsulates the core features of the method. For practitioners of FMD, the muscle test is a central tool for diagnosis and assessment. It enables the therapist or physician to evaluate the neuromuscular response of a muscle subjected to an additional load during maximal voluntary isometric contraction. In this way, the individual’s adaptive capacity can be tested, and potential dysfunctions can be identified. Therefore, the FMD muscle test may serve as a valuable tool for identifying various neuromuscular dysfunctions, including those contributing to the global burden of motor neuron disease [6].

Building on the foundational muscle testing work by Lovett [7], Kendall and Kendall further refined the method by focusing on the maximum force exerted during testing [8]. Goodheart and Walther [9] were the first to emphasize the functional aspects of neuromuscular regulation. Subsequent detailed descriptions of muscle testing procedures and their underlying neurophysiological mechanisms were provided by Gerz [10], Garten [11], and Cuthbert and Goodheart [12].

Muscle responses are traditionally graded on a scale from 0 (no activation) to 5 (full activation against the examiner’s resistance) [13]. In FMD, a grade 5 muscle—provided it shows no weakening or positional shift when subjected to additional force—is considered “strong” or “activated.” However, if compensatory movements or weakening occur under this added load, the muscle is classified as “weak” or “inhibited.” Unlike traditional grading systems, FMD does not use numerical scores; it uses qualitative terms to describe muscle response.

**Figure 1:**
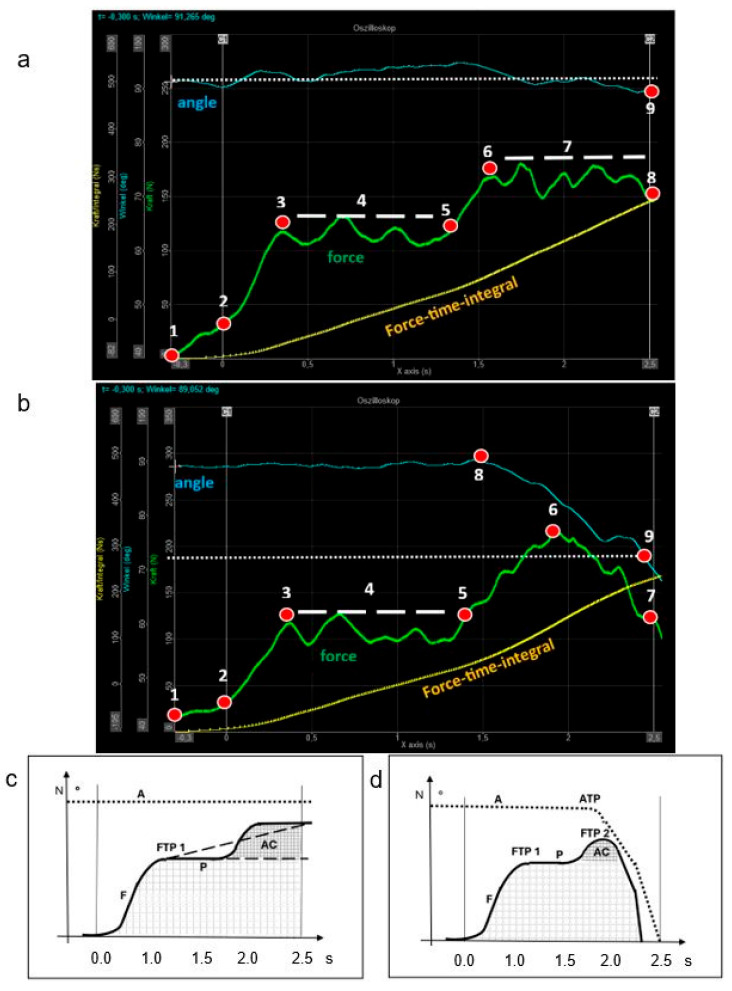
Oscillograms (**a**,**b**) and corresponding schematic diagrams (**c**,**d**) presenting the force (**F**) (Newton (N), y-axis) and joint angle (**A**) curves over time (seconds (s), x-axis) during an FMD muscle test. In strong muscles (**a**,**c**), the force curve (green line) rises sharply, reaches a plateau (force turning point 1), and increases further when the examiner applies additional pressure (additional force load), while the joint angle (blue line) remains stable throughout, reflecting an intact adaptive capacity (**AC**). In contrast, weak muscles (**b**,**d**) show a slower force increase followed by a decline, along with a drop in joint angle—resulting in a lower force–time integral (yellow line), presenting a reduced adaptive capacity. The measurement begins at Cursor 1 (**C1**) at 0.00 s and ends at Cursor 2 (**C2**) at 2.50 s. **1** = Start at the force baseline. **2** = Start of automatic measurement after exceeding a force increase of 30 Newtons. **3** = Force turning point 1 (FTP 1) after the subject reaches the maximum voluntary isometric contraction (concentric phase). **4** = Plateau phase (P) after reaching maximum voluntary contraction. **5** = Beginning of the examiner’s additional pressure applied to the maximally contracted muscle. **6** = Force turning point 2 (FTP 2) after reaching a new force plateau. The increase from point 5 to 6 reflects the additional pressure by the examiner (eccentric phase in the test muscle) on the already maximally contracted test muscle. **7a** = Load plateau phase from point 6 until the end of the measurement period. **7b** = Force level of a weak muscle at the end of the measurement period, followed by a rapid drop-off. **8a** = Force level of a strong muscle at the end of the measurement period. The force–time integral represents the area under the green force line between point 2 and C2. **8b** = Angle Turning Point 1 (ATP 1). **9** = Angle measurement point at the end of the measurement period after 2.50 s.

A correct and reproducible FMD muscle test requires precise positioning of the muscle being tested, with the origin and insertion brought as close as possible. During the initial phase of the test, the patient pushes against the examiner’s hand, increasing force until a plateau is reached (force turning point 1)—representing the peak of voluntary isometric contraction. In the second phase, the examiner applies additional pressure (additional force load) [10,11]. If the muscle maintains or even increases its force output and the testing angle (joint angle) remains stable throughout, this indicates a strong muscle reaction. Such sufficient neuromuscular adaptation of muscle fibers reflects intact compensatory capacity (see Figure 1a,c) [14]. Conversely, if the patient cannot reach a force plateau or cannot withstand the added pressure—resulting in muscle lengthening or deviation from the original position—the force and angle measurements decline, resulting in a reduced force-time integral. This indicates poor recruitment of additional muscle fibers and diminished regulatory capacity, which are all hallmarks of a weak muscle reaction (see Figure 1b,d).

The diagnosis of a weak muscle is unambiguous and is referred to as hypo-reactive. In contrast, a strong muscle can be further categorized as either normo-reactive or hyper-reactive. Normo-reactivity is characterized by a strong muscle response in the absence of any sedating stimulus and a subsequent weak response when a sedating stimulus is applied. Conversely, if a strong muscle does not exhibit an inhibitory response to a sedating stimulus, it is classified as hyper-reactive. Both hypo-reactive and hyper-reactive muscle responses are considered dys-reactive patterns and are classified as pathological within the framework of Functional Myodiagnosis (FMD).

In FMD, the most commonly used sedating stimulus involves the stimulation of specific acupuncture points known to have a sedating effect on particular muscles [10,11,15]. This phenomenon is explained by the interaction between the activation levels of the gamma motoneurons and alpha motoneurons. All neural impulses from the cerebrum, cerebellum, brainstem, and peripheral nerves converge on the alpha and gamma motor neurons in the anterior horn of the spinal cord. The summation of excitatory and inhibitory inputs determines whether the muscle responds as strong or weak [16].

The classification of muscle strength as strong or weak is based on the examiner’s subjective assessment, which has been a major point of criticism. The lack of objective criteria, as required by scientific standards, has limited broader acceptance of FMD in the medical community. As a result, various efforts have been made to objectify the muscle testing procedures used in Applied Kinesiology (AK), as reviewed by Cuthbert and Goodheart [12]. Although some studies have methodological limitations, they have demonstrated moderate to good correlations between clinical assessments and objective measurements [8,9,17,18].

Still, conventional medicine raises significant concerns about Applied Kinesiology (AK), citing a lack of evidence supporting its effectiveness, validity, and reliability. Research in this area often suffers from poor reporting quality. To determine any potential clinical value, AK practitioners need to define clear criteria for its various subgroups and rigorously demonstrate the efficacy of their approaches [19,20].

Thus, the aim of this Muscle Test Objectification study (MTO study) is to investigate if the subjective findings of strong and weak muscle responses in the FMD testing procedure can be objectively and reproducibly verified using quantifiable physical parameters. Further, the present study aims to evaluate the reliability of the manual muscle test and strengthen the scientific evidence for an accepted but not widespread complementary medicine method.

## 2. Materials and Methods

### 2.1. Study Participants

Twenty-four volunteers were recruited in June 2018 from the patient registry of the FMD Research Centre in Kiens. Inclusion criteria included age ≥ 18 years, legal contractual capacity, and the absence of acute major physical or psychological illness. Exclusion criteria were the presence of neuromuscular deficits, any disease or symptoms affecting the hip or knee region, and the presence of dys-reactive muscle responses (hypo- or hyper-reactivity) during the initial examination.

### 2.2. Muscle Test Setup

The muscle evaluated in the MTO study was the right *musculus rectus femoris*, hereafter referred to as the rectus muscle. Testing was performed with the subject in a supine position, which provided both relaxation and stability, as well as optimal access for the clinician. Like other quadriceps muscles, the rectus femoris functions as a knee extensor; however, it uniquely also acts as a hip flexor. In the standardized testing position according to FMD or AK protocols, the rectus muscle’s force vector is mechanically favored over its synergists, making it the primary agonist in the hip flexion movement. This test position included 0° femoral rotation, 90° flexion at the hip, and 80° to 90° flexion at the knee joint [5,8,9,10,11,12].

For this study, the muscle test was conducted on a custom-designed therapy table. The table’s height could be adjusted hydraulically to suit the examiner’s preference. Unlike a standard examination table, the surface was modified to include a freely gliding upholstered platform that moved longitudinally on ball bearings. A force transducer (Type C9C/10 kN, Hottinger Baldwin Messtechnik GmbH, Vienna, Austria) was installed between the gliding platform and the fixed base frame to measure thrust in the direction of the foot end. A restoring mechanism returned the gliding surface to its original position after each test.

**Figure 2:**
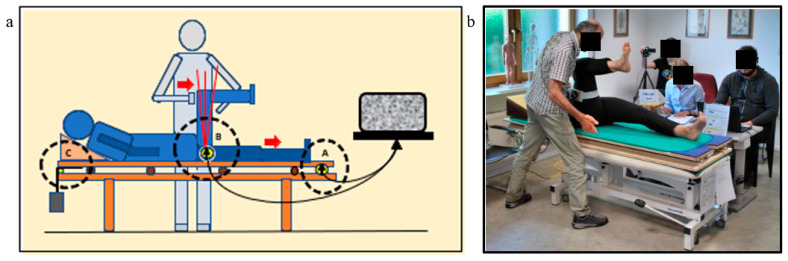
Schematic representation of the FMD muscle test: theoretical setup (**a**) and practical execution (**b**) on the right rectus femoris muscle. The subject lies in a supine position on a custom-designed therapy table. The upper horizontal surface can slide freely along its length on ball bearings (lower red arrow), allowing pure force transmission of the rectus muscle’s horizontally directed force vector (upper red arrow). A force transducer (**A**) is mounted between the sliding board and the fixed base to measure force directed toward the foot end. A restoring mechanism (**C**) repositions the upper section of the table after each test. An electronic goniometer (**B**), or inclinometer, is attached to the subject’s thigh to measure hip joint angles with a precision of 1°. Both sensors are connected to the computer, where the measurement software processes the data in real time. In the practical setup (**b**), the inclinometer is clearly visible on the subject’s right thigh, while in the background, the technical assistant, video operator, and secretary can be seen.

To accurately measure hip angle, an electronic inclinometer (SIKO Inclinometer IK360L, LEOTEC GmbH, Linz, Austria) was affixed to the subject’s thigh, providing angular measurements accurate to 1 degree (see Figure 2). According to the laws of physics, work is defined as the energy transferred through the application of force over a distance. Since the setup prevented any significant displacement of the therapy surface, the muscle’s work could be determined by calculating the force–time integral from the force sensor data.

The outputs from both the force transducer and the inclinometer were processed using dedicated measurement software (DEWESoft X2, Version SP8, DEWESoft GmbH, Kumberg, Austria). The resulting oscillograms allowed for the evaluation of key physical parameters—such as joint angle changes, force–time integral, force turning point 1, and additional applied force—with the aid of the measurement software.

### 2.3. Muscle Test Procedure

**Figure 3:**
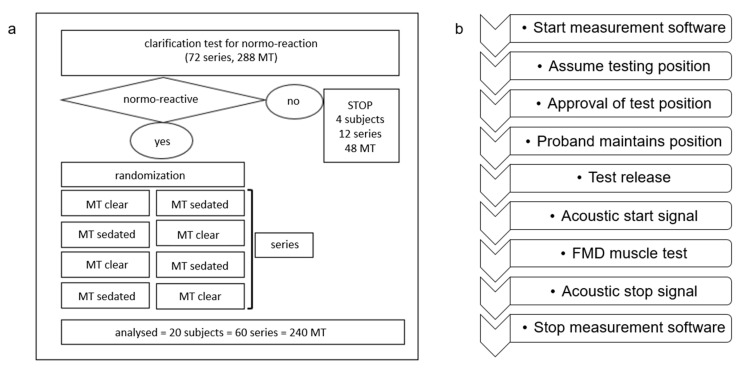
Schematic overview of the FMD muscle test (MT), showing (**a**) the setup and (**b**) the testing procedure.

Prior to testing, all participants underwent an initial screening to confirm grade 5 muscle strength and normo-reactivity according to FMD criteria. The sedating stimulus was administered by activating the small intestine meridian point 8 (SI8), performed by the subjects themselves, following standardized instructions. Therefore, participants were instructed to place the index, middle, and ring fingers of their left hand on their right SI8 point before initiating the muscle test and to maintain this position until the test was completed. Each participant underwent identical testing procedures conducted by three experienced and calibrated examiners (EB, IR, JM), with each examiner performing four tests on the right rectus femoris muscle. These standardized tests were conducted in a randomized sequence of either “clear” (unsedated) or “sedated” muscle conditions. The examiners were blinded to the condition due to the sedation protocol implemented with the subjects (see Figure 3). Additionally, participants rated their general fitness on a visual analogue scale (VAS) ranging from 1 (very poor) to 10 (excellent).

Before testing, participants removed their shoes and all items from their pockets that might interfere with the measurement process. Special attention was paid to ensure that no electronic devices (e.g., mobile phones, keys) were in proximity to the measuring equipment. The correct testing posture for the right rectus femoris, as described above, was carefully established. An experienced FMD physician (RG) supervised the examination procedure, correcting positioning or contact if necessary. The technical assistant (UG) monitored the hip angle in real time on the computer screen and confirmed proper alignment.

The subject was then instructed to actively maintain the testing position while the clinician released the positioning contact. Only the examiner’s left hand remained in contact, placed on the anterior aspect of the distal femur, proximal to the patella. Once the position was secured, the technical assistant initiated the measurement process, signaled by an acoustic cue. The examiner instructed the subject to pull the knee upward toward the head with maximum voluntary effort. Simultaneously, the examiner applied counter-pressure with the left hand to the distal femur. As soon as the force detected by the sensor exceeded 30 Newtons, the software triggered a 3 s measurement window. This threshold prevented premature initiation of the test due to minor, involuntary movements. In the second phase of the muscle test, once the subject had achieved maximum voluntary isometric contraction, the examiner increased force against the femur. The test period ended after 3 s, marked by a second acoustic signal (see Figure 3). After each muscle test, the leg was set down for one minute. Between assessments by different examiners, each subject received a 15 min break. These rest periods were implemented to minimize the risk of fatigue effects influencing the results.

To prevent any bias, examiners had no access to the measurement data or real-time screen recordings during the test. A study coordinator (AS) maintained a secure record linking the examiner and subject IDs with the test type (clear or sedated) based on the randomization protocol. A separate record was kept for video documentation, including the identification of both examiner and participant. For statistical analysis, the start of the measurement window was defined as the point at which the force exceeded 30 Newtons (0.0 s), and the cut-off was set at 2.5 s, or earlier if the hip angle deviated more than 15° from its baseline.

### 2.4. Statistical Methods

For the primary endpoint force-time integral, a power analysis estimated that a minimum of 12 participants would be needed to detect an effect size of 1.22 with 80% power (α = 0.05, two-tailed). This estimate was based on pilot data showing a within-subject mean difference of 50 units and a standard deviation of 41. To account for a 25% dropout rate, the sample size was increased to 15. To enhance the credibility of the results, the final sample size was expanded to 24 participants. Mean group differences are expressed using Cohen’s d, where effect sizes of 0.1, 0.4, and 0.8 are interpreted as small, medium, and large, respectively [21].

Data analysis was conducted using SPSS for Windows, Version 30.0 (SPSS Inc., Chicago, IL, USA). Descriptive statistics were used to verify that assumptions for inferential tests were met. Continuous variables are reported as means and standard deviations or medians and ranges, as appropriate. Categorical data are presented as frequencies and percentages. Frequencies were compared using the Chi-square test. To analyse continuous variables and account for repeated measures within subjects (Latin square design), a linear mixed-effects model was applied, with fixed effects for test sequence, test condition (strong/weak), sex, age, BMI, and the interaction between rater and test. The validity of the clinical and instrumental FMD muscle test evaluations was assessed using the Chi-square test. Inter-rater reliability was calculated using the Intraclass Correlation Coefficient (ICC 2.1A) for a two-way random-effects model, single measure, with absolute agreement [22]. An ICC value < 0.5 was interpreted as poor, 0.5 to 0.75 as moderate, 0.75 to 0.9 as good, and 0.9 as excellent reliability. The differentiation of key physical parameters—averaged across all examiners—including change in joint angle, force–time integral, force turning point 1, and additional force load, between strong and weak muscle conditions over the 2.5 s test period, was analyzed using the Wilcoxon signed-rank test. All tests were two-sided, and a *p*-value < 0.05 was considered statistically significant.

## 3. Results

### 3.1. Study Population

Of the initial 24 individuals enrolled, 4 subjects were excluded due to dys-reactive muscle responses observed during the preliminary screening—1 exhibited a hypo-reactive response and 3 showed hyper-reactive responses. Consequently, the final study population consisted of 20 participants (9 males and 11 females; all of Caucasian ethnicity), with a mean age of 51 years (range: 23–71), a mean body mass index (BMI) of 23.88 (range: 18.3–28.4), and a self-reported mean fitness level of 7 on a 10-point visual analogue scale (range: 4–10), indicating an overall moderate level of fitness (see Table 1). Since each participant completed four muscle tests performed by each of the three examiners, a total of 240 muscle tests were available for statistical analysis.

### 3.2. Physical Parameters of the FMD Muscle Test

**Figure 4:**
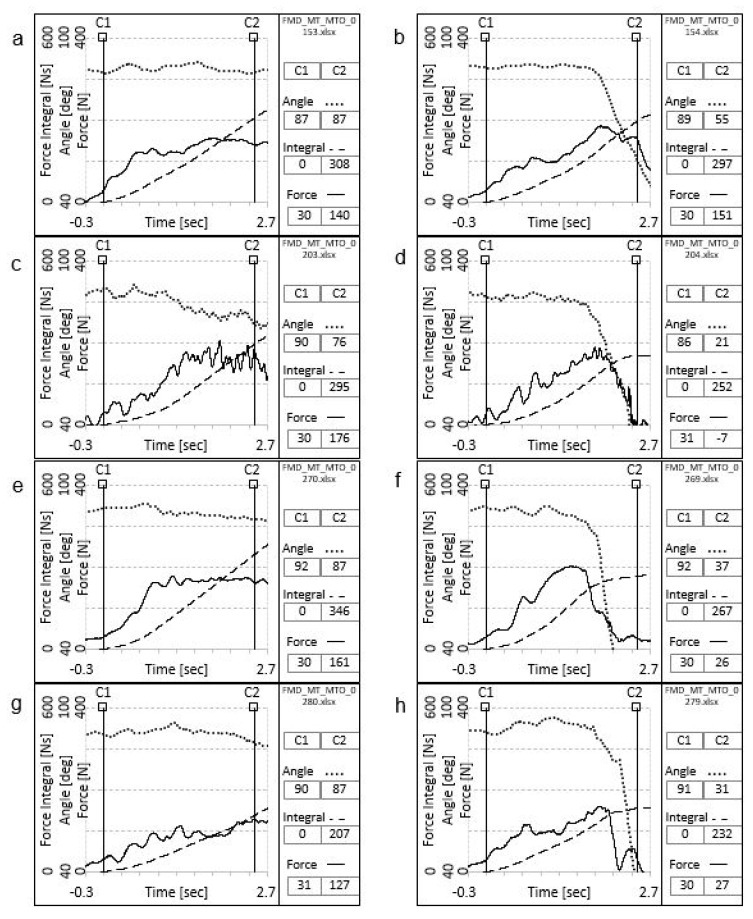
Characteristics of strong and weak muscle responses based on oscillograms from four subjects (**a**–**h**), with force (N, Newton; y-axis) plotted over time (sec, seconds; x-axis) from 0 (**C1**) to 2.5 s (**C2**). In the case of strong muscles (**a**,**c**,**e**,**g**), the force curve (**F**, solid line) remains stable despite the application of an additional load, and the angle curve (A, dotted line) exhibits minimal deviation only. This pattern reflects a well-preserved adaptive capacity. Conversely, weak muscles (**b**,**d**,**f**,**h**) display an initial increase in force followed by a decline in both force and angle upon load application, indicating inadequate adaptation to the external load. Dotted line = joint angle (deg, degrees); dashed line = force–time integral (Ns, Newton seconds).

Distinct differences in response patterns between strong and weak muscles were evident in the oscillographic recordings, with characteristic patterns illustrated in Figure 4. All physical parameters were derived from the mean values observed for the strong and weak muscle test conditions (see Table 2).

The mean force–time integral for strong muscles was 264 Ns (range: 174–328 Ns), while for weak muscles it was 237 Ns (range: 147–321 Ns). This difference was statistically significant (*p* = 0.005).

The mean change in joint angle was 9.4° (range: 7.2–16.8°) for strong muscles, and 38.2° (range: 13.7–59.0°) for weak (inhibited) muscles. This difference was also statistically significant (*p* < 0.001).

The mean time to force turning point 1 was 1.0 s for both strong (range: 0.10–2.50 s) and weak muscles (range: 0.20–2.40 s), with no significant difference between the two conditions (*p* = 0.972).

The mean additional force load applied by the examiner was 29.3% (range: 7.0–60.9%) for strong muscles, compared to 45.9% (range: 22.5–87.5%) for weak muscles. This difference was statistically significant (*p* = 0.001).

### 3.3. Validity of the Clinical Analysis of the FMD Muscle Test

The overall validity of the examiners’ clinical interpretation of muscle response, when compared to the actual test condition (sedated or non-sedated), was 99.6%. Examiners correctly identified 100% (95% CI, 97.9–99.9) of weak muscle reactions and 99.2% (95% CI, 96.2–99.9) of strong muscle reactions. There was no statistically significant difference in diagnostic accuracy between strong and weak muscle conditions (*p* = 0.316).

### 3.4. Interrater Reliability of the FMD Muscle Test

The overall intra-class correlation coefficient (ICC) for the classification of muscle strength (strong vs. weak) was 0.984 (95% CI, 0.86–1.11), indicating excellent interrater reliability, as defined by Koo and Li [22].

### 3.5. Validity of the Instrumental Analysis of the FMD Muscle Test

The overall validity of the interpretation of the oscillogram at 2.5 s, compared to the actual muscle test condition (sedated or non-sedated), was 84.2%. The oscillogram correctly represented a weak muscle reaction in 81.7% (95% CI, 74.0–87.8) of cases and a strong muscle reaction in 86.7% (95% CI, 79.7–91.9) of cases. No significant difference in validity was observed between strong and weak conditions (*p* = 0.289).

## 4. Discussion

The aim of this study was to clarify the evidence of the complementary medicine method Functional Myodiagnosis (FMD) regarding its validity, reliability, and objectivity.

The rectus muscle was chosen in the present study for the test procedure of Functional Myodiagnosis muscle testing (FMD-MT), because it is repeatedly well testable, and the sedation point is easily accessible. Further, due to the horizontal force vector, it is the ideal muscle for the specially-designed therapy bench. The activation of the small intestine meridian point 8 (SI8) by the subject was the sedation stimulus, which was chosen in this trial. The authors intensively discussed this topic, but as all alternative sedation methods (approximation of the muscle, sedation with magnet, running against meridian, or using other sedating acupuncture points) would have made the test procedure much more complicated, there was a clear agreement on SI8.

Given the relatively small sample size of 20 subjects, small effects may have remained undetected. However, since each participant underwent four muscle tests conducted by three different examiners, a total of 240 muscle tests were available for statistical analysis, allowing for robust results. A parallel-group or cohort design offers many advantages but would not have been feasible in this study, as it was important to repeatedly test both variants—strong and weak—within the same individual. Randomization was performed prior to the examination. All examiners were blinded to the randomized sedation procedure, so that the concurrence between examiner and oscillogram to the real proceeded muscle test could be analyzed unadulteratedly. A double-blind study design is not possible, as the probands will quickly realize the influence of the stimulus set on their body.

The specially designed therapy couch with its freely gliding surface board was of great help. Unlike most studies that have been undertaken to date, it offers the unique possibility to examine the patient in everyday conditions. Generally, in muscle force studies, handheld dynamometers are used [23,24]. This means that there is an artificial contact between the clinician and the patient, which can be imprecise and irritating. Even when thin film force transducers are at hand, the reliability may be strongly reduced because of the contact with a small part of the palm of the hand only. This was not the case in this study, since the examiner/subject contact was not impaired. However, this therapy table design is restricted to the muscles of the human body, which results in a strictly horizontal and caudally directed force vector only.

This study has several important limitations that should be addressed. The small sample size and the exclusive inclusion of normo-reactive participants—excluding both hypo- and hyper-reactive individuals—limit the generalizability of the findings to broader populations. Additionally, the sample lacks ethnic diversity and is skewed toward an older average age (mean 51 years), further restricting external validity. The short recording window and reliance on subjective concepts may also impact the robustness of the results. Finally, the clinical applicability of the findings remains uncertain, as practical implementation challenges have not yet been explored. Future research should aim to include larger, more ethnically diverse, and age-varied samples with a range of muscle reactivity profiles, as well as longer observation periods of different muscles, to better validate and translate the method into clinical practice.

Clear graphical correspondence between strong and weak muscle reactions, as defined in FMD, could be demonstrated. This was shown by the significant (*p* < 0.001) change in joint angle as a major criterion of the test result, which permits objectification of the subjective findings of the clinicians in the strong and weak test variants. Strong muscles correlate with successful adaptation to additional load with constant joint angle of median 9.4° (range: 7.2–16.8°), while weak muscles show a lack of adaptation in response to additional force load with relevant change in joint angle of median 38.2° (range: 13.7–59°). It is noteworthy that Bittmann [25] recorded angle patterns that are almost identical to those observed in the MTO study. While strong or stable muscles show no yielding when an additional load is applied, weak or unstable muscles can be identified by the displacement of the limb, even though the maximum force is not reached. However, due to differences in study design, a direct comparison with the MTO study is not possible. The primary goal of Bittmann and Schaefer’s work was to objectify subjective clinical findings through the development of a pneumatic measurement system [26]. In contrast, the MTO study maintained typical clinical contact with the patient, while objectification was achieved using a force transducer embedded in the therapy couch.

The force–time integral differed significantly between the two test variants (*p* = 0.005), with values of 264 Ns (range: 174–328 Ns) for strong muscles and 237 Ns (range: 147–321 Ns) for weak muscles. This result is crucial when using the body’s muscular system as a diagnostic tool, as it provides clinicians with a reliable and practical method for making individualized treatment decisions [27,28]. It allows for a clear distinction between weak and strong muscles, enabling the identification of both strengthening and weakening therapeutic stimuli.

In our trial, no significant difference was found between strong and weak muscle reactions regarding the mean force turning point 1 (*p* = 0.972). Both groups reached this point at an average of 1.0 s (strong range: 0.10–2.50 s; weak range: 0.20–2.40 s). This parameter is a key factor in everyday clinical practice for performing an accurate muscle test. The force turning point can be perceived by the examiner as a plateau, indicating the precise moment when additional force should be applied. The speed at which a subject reaches this point depends on their level of training—more trained individuals typically reach it faster. Given that the participants had an average fitness level of 7, a mean duration of 1 s is a plausible and clinically meaningful result.

In this MTO study, the mean additional force load following the maximum voluntary isometric contraction was 29.3% (range: 7–60.9%) on strong, and 45.9% (range: 22.5–87.5%) on weak muscles, with a significant difference between the variants (*p* = 0.001). Gerz [10] and Goodheart/Garten [11] quoted an additional force load exerted by the clinician in order to evaluate the adapting ability of only up to 4%. Burtscher [1,3] postulated a noticeably higher additional force load of approximately 30%. Similar results to our outcome were reported in sports science [14] and in preliminary pilot studies (unpublished data), where the additional force was predominantly 30–50%.

As shown in numerous studies by Bittmann and Schaefer, even when weak muscles yield, force output can still increase [26,27]. Eccentric muscle force must exceed concentric force to serve as a protective mechanism for the body, such as during stumbling or decelerating movements in sports. To better describe this aspect of neuromuscular function, the term “adaptive force” was introduced in 2015 [29]. From this perspective, it is reasonable to observe an ascending force curve even in weak or unstable muscles, while the joint angle curve is already declining—indicating that the limb is initially giving way. When there is minimal overlap between myosin and actin filaments, the force curve also drops rapidly [16], see Figure 1d.

Recent neuromuscular control models emphasize coordinated α-γ motoneuron activity, dividing motor commands into static and dynamic parts to regulate joint position and stiffness [30]. The γ system modulates proprioceptive sensitivity and timing of antagonist muscles during movement. Our study supports these ideas: weak muscles showed greater joint angle changes and lower force–time integrals, indicating reduced neuromuscular stability, possibly due to impaired α-γ integration or feedback loops. These findings align with current theories of α-γ coordination affecting motor control and proprioception.

In this trial, the total validity of the examiner’s interpreted results of muscle reaction was 99.6% (100% for weak, 99.2% for strong muscles). A review by Cuthbert reports validity of up to 99%, depending on the muscle tested and the examiner’s level of experience [12]. Still, it must be considered that this trial was conducted under perfect conditions, that only normo-reactive probands were included, and that the probands got enough time to rest between the testing sequences, which only contained four muscle tests from each examiner.

An excellent interrater reliability [22] of the FMD muscle test for the rectus muscle with an ICC of 0.984 could be demonstrated in this trial. These results are comparable to a study by Caruso and Leisman, which demonstrated a reliability of 98.2% for the AK muscle test among clinicians with more than five years of experience [31]. Previous studies reported different reliability for different muscles. For example, good interexaminer reliability for the psoas and the deltoid muscle [32] and for the piriformis and the pectoralis muscle, but little or no significant concordance was found for the hamstrings and the tensor fascia lata muscle test, respectively [23].

The overall validity of the interpreted oscillograms after 2.5 s, compared to the actual muscle test condition (sedated or not), was 84.2% (81.7% for weak muscles, 86.7% for strong muscles; *p* = 0.289). One possible reason the oscillograms did not show higher validity is that the recording time window was too short. We propose that a longer test duration would likely improve the ability to differentiate between weak and strong muscles using oscillograms. Supporting this, in a pilot study using thin-film force transducers, K. Conable demonstrated that longer test durations are preferable for identifying weak muscles [33]. Future studies should aim to extend the recording duration beyond 2.5 s and apply advanced analytical methods, including machine learning, to enhance the precision of oscillogram analysis.

The characteristics of muscle testing are described by many AK and FMD therapists worldwide. The MTO study illustrates the biophysical sequences of the FMD muscle test (FMD-MT) in various ways and provides clear key statistical data. However, this study is restricted to the rectus femoris muscle, which narrows the generalizability of the findings. Given the increasing interest in FMD muscle testing, it is crucial to investigate a wider range of muscles with varied biomechanical properties to deepen the understanding of neuromuscular adaptability in grade 5 muscles, to strengthen methodological validation, and also to enhance potential clinical applications in rehabilitation, sports science, and geriatric functional assessments. Considering the current therapy table design, future studies should examine the tibialis anterior and serratus anterior in the supine position, as well as the hamstrings in the prone position. Moreover, assessing the validity and interrater reliability of these muscles will be essential to confirm the robustness of the FMD muscle test.

## 5. Conclusions

In summary, under rigorously standardized testing conditions, the Functional Myodiagnosis (FMD) muscle test applied to the rectus femoris muscle exhibited a validity rate of 99.6% and demonstrated excellent reliability, with an intraclass correlation coefficient (ICC) of 0.984. It allows for a clear and objective distinction between strong and weak muscles, with statistically significant differences observed in the force-time integral, additional force load, and joint angle changes. Therefore, the FMD muscle test of the rectus femoris muscle appears to be a reliable, reproducible, and objective diagnostic tool.

## Figures and Tables

**Table 1:** Distribution of age, sex, ethnicity, body mass index, and fitness level.

**Age (Years)**	Mean (Range)	51 (23–71)
	male	9
**Sex**	female	11
	others	0
**Ethnicity**	Caucasian	20
**BMI**	Mean (range)	23.88 (18.3–28.4)
**Fitness level**	Mean (range)	7 (4–10)

**Table 2:** Results of key physical parameters and validity measures for strong and weak muscle reactions. Ns = Newton seconds, ° = degrees, s = seconds, % = percent, CI = confidence interval, *p* = *p*-value.

	Strong	Weak	*p*-Value	Cohen’s d
**Force–time integral, Ns**	264 ± 60.8 95% CI, 253.0–275.0 Range, 174–328	237 ± 68.1 95% CI, 224.7–249.3 Range, 147–321	*p* = 0.005	0.64
**Change in joint angle, °**	9.4 ± 5.3 95% CI, 8.4–10.3 Range, 7.2–16.8	38.2 ± 19.2 95% CI, 34.7–41.7 Range, 13.7–59.0	*p* < 0.001	3.69
**Force turning point 1, s**	1.0 ± 0.5 95% CI, 0.9–1.1 Range, 0.10–2.50	1.0 ± 0.5 95% CI, 0.9–1.1 Range, 0.20–2.40	*p* = 0.972	0.00
**Additional force load, %**	29.3 ± 31.0 95% CI, 23.7–34.9 Range, 7.0–60.9	45.9 ± 34.5 95% CI, 39.7–52.2 Range, 22.5–87.5	*p* = 0.001	0.99

## Data Availability

The datasets used and/or analyzed during the current study are available from the corresponding author on reasonable request.
